# Supplementary material for: Time-Optimal Adaptation in Metabolic Network Models
Source: Front Mol Biosci. 2022 Jul 14;9:866676. doi: 10.3389/fmolb.2022.866676 (PMC9329932; doi:10.3389/fmolb.2022.866676)
Supplement: Supplementary file 1 [file DataSheet1.pdf]

Supplementary Material

S1 ABBREVIATIONS AND NOTATIONS

$\boldsymbol{y}(t)$	total molecular amounts (of metabolites) within the cell
$\boldsymbol{v}(t)$	flux rates in the metabolic network
S	stoichiometric matrix
$\boldsymbol{u}(t)$	collection of (mostly time-dependent) degrees-of-freedom in the network (mostly flux rates)
$\dot{(\cdot)}$	time derivative, e.g. $\dot{\boldsymbol{y}}(t) = \frac{\mathrm{d}}{\mathrm{d}t}\boldsymbol{y}(t)$
$\boldsymbol{w}$	vector of molecular weights
$\mathit{bio}(t)$	biomass $\boldsymbol{w}^\top \cdot \boldsymbol{y}(t)$ of the cell
$\boldsymbol{c}(t)$	concentrations of metabolites $\boldsymbol{c} = \frac{\boldsymbol{y}}{\boldsymbol{w}^\top \cdot \boldsymbol{y}}$
$\mathbf{0}, \mathbf{0}$	zero vector, zero matrix
$[t_0, T^*]$	time interval of interest
$\mathcal{A}(\cdot)$	(abstract) set defining the constraint-based modeling framework, see **(2.7c)**
λ	Growth rate of the cell (given exponential growth from mass m_0 to mass m_1 in a time interval of length Δ: $\lambda = \frac{\ln(m_1/m_0)}{\Delta}$)
$(\cdot)^\top$	transpose of a vector/matrix
$(\cdot)_{i_1:i_2}$	indexing of a vector: For $\boldsymbol{a} = (a_1, a_2, \ldots, a_n)^\top$ and positive integers $i_1 \leq i_2$, it holds $\boldsymbol{a}_{i_1:i_2} := (a_{i_1}, a_{i_1+1}, \ldots, a_{i_2})^\top$. If no start/end index is supplied, all remaining indices are included: $\boldsymbol{a}_{i_1:} := (a_{i_1}, a_{i_1+1}, \ldots, a_n)^\top$
$\leq$	(component-wise) smaller-or-equal relation
LP	Linear program/linear programming
TOA(-VA)	time-optimal adaptation (Variability Analysis)
FBA	flux balance analysis
FVA	flux variability analysis
cFBA	conditional FBA
RBA	resource balance analysis
deFBA	dynamic enzyme-cost FBA

S2 SELF-REPLICATOR MODEL

S2.1 Model Description

Compounds: The vector of metabolic compounds is given by $\boldsymbol{y}(t) = (M(t), Tr(t), R(t))^\top$; additionally, there is an external nutrient supply of total amount $N(t)$ that is controlled by the external conditions.

Reactions: The vector of reaction fluxes is given by $\boldsymbol{u}(t) = (v_N(t), v_{Tr}(t), v_R(t), vd_{Tr}(t), vd_R(t))^\top$ with the reactions

- $v_N : N \xrightarrow{Tr} M$
- $v_{Tr} : (n_{Tr} - n_{Tr,\mathrm{red}}) \cdot M \xrightarrow{R} Tr$
- $v_R : (n_R - n_{R,\mathrm{red}}) \cdot M \xrightarrow{R} R$
- $vd_{Tr} : Tr \rightarrow n_{Tr} \cdot M$
- $vd_R : R \rightarrow n_R \cdot M$

Constraints:

1. Positivity: $M(t), Tr(t), R(t) \geq 0$
2. Irreversibility: $v_N(t), v_{Tr}(t), v_R(t), vd_{Tr}(t), vd_R(t) \geq 0$
3. Dynamics:

$$\begin{aligned}
\dot{M}(t) &= v_N(t) - (n_{Tr} - n_{Tr,\mathrm{red}}) \cdot v_{Tr}(t) - (n_{R,\mathrm{red}} - n_R) \cdot v_R(t) + n_{Tr} \cdot vd_{Tr}(t) + n_R \cdot vd_R(t) \\
\dot{Tr}(t) &= n_{Tr,\mathrm{red}} \cdot v_{Tr}(t) - n_{Tr} \cdot vd_{Tr}(t) \\
\dot{R}(t) &= n_{R,\mathrm{red}} \cdot v_R(t) - n_R \cdot vd_R(t)
\end{aligned}$$

4. Enzyme Capacity constraints:

$$v_N(t) \leq \frac{N(t)}{1 + N(t)} \cdot k_{\mathrm{cat},Tr} \cdot Tr(t)$$

$$\frac{n_{Tr}}{k_{\mathrm{cat},Tr}} \cdot v_{Tr}(t) + \frac{n_R}{k_{\mathrm{cat},R}} \cdot v_R(t) \leq R(t)$$

Constants:

$$\begin{aligned}
n_{Tr} &= 10\,, & n_R &= 15\,, \\
n_{Tr,\mathrm{red}} &= (1-\beta) n_{Tr}\,, & n_{R,\mathrm{red}} &= (1-\beta) n_{Tr}\,, \\
k_{\mathrm{cat},Tr} &= 10\,, & k_{\mathrm{cat},R} &= 10\,, \\
\beta &= 0.2\,,
\end{aligned}$$

S2.2 Re-formulation as a deFBA problem

In the terms of **Example 2.2**, the dynamics of the self-replicator model can be described via:

$$\mathbf{S}_{\boldsymbol{y}} = \begin{pmatrix} 1 & -(n_{Tr} - n_{Tr,\text{red}}) & -(n_R - n_{R,\text{red}}) & n_{Tr} & n_R \\ 0 & n_{Tr,\text{red}} & 0 & -n_{Tr} & 0 \\ 0 & 0 & n_{R,\text{red}} & 0 & -n_R \end{pmatrix} \in \mathbb{R}^{3\times 5}\,,$$

$$\mathbf{S}_{\boldsymbol{x}} = \mathbf{0} \in \mathbb{R}^{0\times 5}\,,$$

$$\boldsymbol{lb} = \mathbf{0} \in \mathbb{R}^5\,, \quad \boldsymbol{ub}_i = +\infty \text{ for } i = 1,2,3,4,5,$$

$$\mathbf{H}_{\boldsymbol{y}}(t) = \begin{pmatrix} 0 & \frac{-N(t)}{1+N(t)} \cdot k_{\text{cat},Tr} & 0 \\ 0 & 0 & -1 \end{pmatrix} \in \mathbb{R}^{2\times 3}\,,$$

$$\mathbf{H}_{\boldsymbol{v}}(t) = \begin{pmatrix} -1 & 0 & 0 & 0 & 0 \\ 0 & \frac{-n_{Tr}}{k_{\text{cat},Tr}} & \frac{-n_R}{k_{\text{cat},R}} & 0 & 0 \end{pmatrix} \in \mathbb{R}^{2\times 5}\,,$$

$$\boldsymbol{h}(t) = \mathbf{0} \in \mathbb{R}^2\,.$$

S2.3 Formulation including Extracellular Compound Dynamics

In the above formulation of TOA, extracellular compounds (like nutrients or waste products) are not explicitly incorporated into the framework and all dynamic evolution (by means of ODEs) is restricted to the total cellular amounts $\boldsymbol{y}(t)$. The influence of the environment to the cell is captured by the abstract constraint set $\mathcal{A}(t)$ in the general description **(2.3)** or, more specifically, by the matrices/vectors $\mathbf{H}_{\boldsymbol{y}}(t)$, $\mathbf{H}_{\boldsymbol{v}}(t)$, $\boldsymbol{h}(t)$ in **(2.4)**. If the amounts of extracellular compounds (now denoted by $\boldsymbol{e}(t) \in \mathbb{R}^{n_e}$) are part of the unknowns in the model, the constraint-based framework **(2.3)** can be adapted to include dynamic relations, i.e., DAEs including $\dot{\boldsymbol{e}}(t)$ as well. Formula **(2.3a)** can then be extended to

$$\text{for (almost) all } t\text{:}\;\; (\dot{\boldsymbol{y}}(t), \dot{\boldsymbol{e}}(t), \boldsymbol{y}(t), \boldsymbol{e}(t), \boldsymbol{u}(t)) \in \tilde{\mathcal{A}}(t)\,, \tag{2.3a*}$$

with an appropriate extension $\tilde{\mathcal{A}}(t)$ of the original constraint set $\mathcal{A}(t)$. All frameworks covered by **(2.3)** can be formulated in terms of **(2.3a*)** as well and, accordingly, TOA can also be formulated with $\boldsymbol{e}(t)$ as part of the dynamic variables. However, we note that providing goal states for external biochemicals is more unrealistic from the modeling viewpoint as the cell only has very limited control over its surroundings.

Instead of re-formulating the frameworks in **Examples 2.1, 2.2, 2.3** and **2.4** in detail, we will just show here (i) how the self-replicator model can be re-framed to include limited total nutrient availability by considering dynamics of $N(t)$ and (ii) how the original model can be recovered again by adapting the flux vector. As the uptake reaction is nonlinear (Michaelis-Menten kinetics), a fully linear model is no longer possible.

(i) Introduce vectors $\boldsymbol{y}(t) = (y_i)_{i=1,2,3} = (M(t), Tr(t), R(t))^\top) \geq \mathbf{0}$, $\boldsymbol{e}(t) = (e_i)_{i=1} = (N(t)) \geq 0$, $\boldsymbol{v} = (v_N, v_{Tr}, v_R, vd_{Tr}, vd_R)^\top \geq \mathbf{0}$, and let

$$\begin{aligned}
\dot{y}_1(t) &= v_N(t) - (n_{Tr} - n_{Tr,\text{red}}) \cdot v_{Tr}(t) - (n_{R,\text{red}} - n_R) \cdot v_R(t) + n_{Tr} \cdot vd_{Tr}(t) + n_R \cdot vd_R(t) \\
\dot{v}_2(t) &= n_{Tr,\text{red}} \cdot v_{Tr}(t) - n_{Tr} \cdot vd_{Tr}(t) \\
\dot{v}_3(t) &= n_{R,\text{red}} \cdot v_R(t) - n_R \cdot vd_R(t) \\
\dot{e}_1(t) &= -v_N(t)
\end{aligned}$$

subject to:

$$\begin{aligned}
v_N(t) &\leq \frac{e_1(t)}{1 + e_1(t)} \cdot k_{\text{cat},Tr} \cdot y_2(t) \\
\frac{n_{Tr}}{k_{\text{cat},Tr}} \cdot v_{Tr}(t) + \frac{n_R}{k_{\text{cat},R}} \cdot v_R(t) &\leq y_3(t)
\end{aligned}$$

and provide initial values for the external nutrient $N(t_0)$. In this formulation, no external nutrient source is present anymore and $N(t)$ gets accordingly depleted over time (for TOA, this would have important implications as the environment the cell adapts to is no longer the same after the adaptation process).

(ii) If the original problem were to be recovered while still including the dynamics of $\boldsymbol{e}(t)$, one can (there are multiple ways to model this) introduce another flux rate $v_{\text{in}}(t) \geq 0$ that compensates the reduction of the nutrient. The differential equation for $e_1(t)$ would then need to be adapted to

$$\dot{e}_1(t) = -v_N(t) + v_{\text{in}}(t)\,, \tag{A.1}$$

and the overall nutrient level could be kept at a given level $N_{\text{given}}(t)$ via (in-) equality constraints

$$N_{\text{given}}(t) \leq e_1(t) \leq N_{\text{given}}(t)\,.$$

Numerically, this re-formulation is (unnecessarily) complex as the differential equation **(A.1)** is merely a dummy equation and a linear problem is formulated in nonlinear terms. We state the alternative form here to show that a more general approach using external compounds $\boldsymbol{e}(t)$ is able to capture more general biochemical questions; the above restriction to dynamics of $\boldsymbol{y}(t)$ was mainly for notational convenience.

S3 FORMULATION IN TERMS OF CONCENTRATIONS

The constraints on the boundary values are given by

1. Initial values (as usual):

$$\boldsymbol{y}(t_0) = \boldsymbol{y}^{\text{init}} \qquad \Rightarrow \quad \mathbf{I}_{n_{\boldsymbol{y}}} \cdot \boldsymbol{y}(t_0) = \boldsymbol{y}^{\text{init}}$$

2. Final values:

$$\frac{\boldsymbol{y}(T)}{\boldsymbol{w}^\top \cdot \boldsymbol{y}(T)} = \frac{\boldsymbol{y}^{\text{goal}}}{\boldsymbol{w}^\top \cdot \boldsymbol{y}^{\text{goal}}} \qquad \Rightarrow \quad \mathbf{B}_{\boldsymbol{y}_{\text{goal}}} \cdot \boldsymbol{y}(T) = \mathbf{0}\,,$$

   where $\mathbf{B}_{\boldsymbol{y}_{\text{goal}}} \in \mathbb{R}^{n_{\boldsymbol{y}} \times n_{\boldsymbol{y}}}$ is defined through

$$\mathbf{B}_{\boldsymbol{y}_{\text{goal}}} = \boldsymbol{y}^{\text{goal}} \cdot \boldsymbol{w}^\top - \left(\boldsymbol{w}^\top \cdot \boldsymbol{y}^{\text{goal}}\right) \cdot \mathbf{I}_{n_{\boldsymbol{y}}}$$

This means, the boundary conditions to be imposed by the optimal control solver are given by

$$\begin{pmatrix} \mathbf{I}_{n_{\boldsymbol{y}}} \\ \mathbf{0} \end{pmatrix} \cdot \boldsymbol{y}(t_0) + \begin{pmatrix} \mathbf{0} \\ \mathbf{B}_{\boldsymbol{y}_{\text{goal}}} \end{pmatrix} \cdot \boldsymbol{y}(t_{\text{end}}) = \begin{pmatrix} \boldsymbol{y}^{(0)} \\ \mathbf{0}_{|\mathcal{J}|} \end{pmatrix}\,.$$

S4 ENZYME FRACTIONS RELATIVE TO GROWTH RATE

The following plot shows the relation between "optimal" molecular amounts (as predicted by RBA) and growth rate of the cell, cf. **Figure 2** and (Scott and Hwa, 2011).

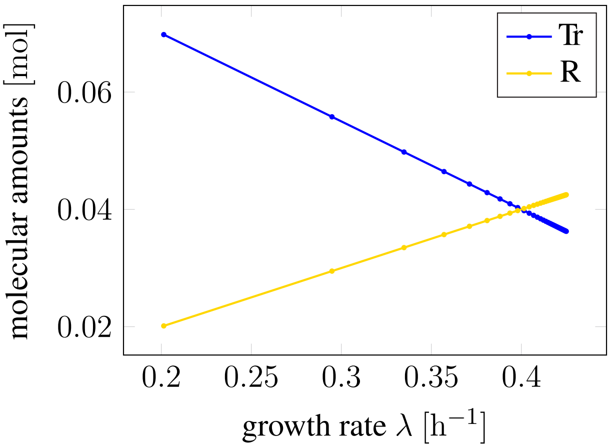

Figure S1. Cellular amounts of intracellular compounds as functions of maximal growth rate. Extracellular nutrient is measured relative to the Michaelis constant K_M of the uptake reaction.
